# Nano-imaging confirms improved apatite precipitation for high phosphate/silicate ratio bioactive glasses

**DOI:** 10.1038/s41598-021-98863-3

**Published:** 2021-09-30

**Authors:** Altair T. Contreras Jaimes, Gloria Kirste, Araceli de Pablos-Martín, Susanne Selle, Juliana Martins de Souza e Silva, Jonathan Massera, Natalia Karpukhina, Robert G. Hill, Delia S. Brauer

**Affiliations:** 1 Otto Schott Institute of Materials Research, Friedrich Schiller University, Fraunhoferstr. 6, 07743 Jena, Germany; 2 Fraunhofer Institute for Microstructure of Materials and Systems IMWS, Walter-Hülse-Str. 1, 06120 Halle, Germany; 3 Institute of Physics, Martin Luther University Halle-Wittenberg, Heinrich-Damerow-Str. 4, 06120 Halle, Germany; 4 Faculty of Medicine and Health Technology, Tampere University, Korkeakoulunkatu 3, 33720 Tampere, Finland; 5 Dental Physical Sciences, Barts and the London School of Medicine and Dentistry, Queen Mary University of London, Mile End Road, London, E1 4NS UK; 6 Leibniz Institute for Solid State and Materials Research Dresden, Helmholtzstr. 20, 01069 Dresden, Germany

**Keywords:** Biomaterials, Glasses

## Abstract

Bioactive glasses convert to a biomimetic apatite when in contact with physiological solutions; however, the number and type of phases precipitating depends on glass composition and reactivity. This process is typically followed by X-ray diffraction and infrared spectroscopy. Here, we visualise surface mineralisation in a series of sodium-free bioactive glasses, using transmission electron microscopy (TEM) with energy-dispersive X-ray spectroscopy (EDXS) and X-ray nano-computed tomography (nano-CT). In the glasses, the phosphate content was increased while adding stoichiometric amounts of calcium to maintain phosphate in an orthophosphate environment in the glass. Calcium fluoride was added to keep the melting temperature low. TEM brought to light the presence of phosphate clustering and nearly crystalline calcium fluoride environments in the glasses. A combination of analytical methods, including solid-state NMR, shows how with increasing phosphate content in the glass, precipitation of calcium fluoride during immersion is superseded by fluorapatite precipitation. Nano-CT gives insight into bioactive glass particle morphology after immersion, while TEM illustrates how compositional changes in the glass affect microstructure at a sub-micron to nanometre-level.

## Introduction

Bioactive glasses are used clinically as granules for bone regeneration, such as in periodontal disease, for filling of alveolar sockets or for treating chronic bone infections^1,2^. Two clinically approved compositions are phospho-silicate glasses 45S5 and S53P4, commercialised as NovaBone® and Bonalive®, respectively. Such bioactive glass compositions usually contain sodium, which was initially added for lowering the melting temperature and long considered the reason for fast glass degradation in contact with aqueous solutions^3^. However, it was later shown that the rapid pH increase caused by large sodium concentrations in a bioactive glass has adverse effects in cell culture testing even in a buffered solution such as cell culture medium^4^. Also, acellular immersion experiments showed that sodium is not really necessary for making glasses degrade, release ions or form an apatite surface layer^5,6^, and that ion release and apatite formation can be maintained in calcium phospho-silicate glasses if the phosphate content is increased without increasing the silicate network polymerisation^7–9^. As removing sodium from a bioactive glass and replacing it with calcium increases the melting temperature, fluoride may be added to the glass, as it effectively lowers the melting temperature^10^, to compensate for the traditional fluxing agent Na_2_O. Besides, fluoride accelerates apatite precipitation by lowering apatite solubility^11^, and fluoride release from bioactive glasses was shown to promote osteoblast proliferation, differentiation and mineralisation in vitro^12^.

Here, we investigate a series of sodium-free glasses with varying phosphate/silicate ratio and reactions. The study of the influence of this compositional ratio is interesting mainly for two reasons: (1) during immersion it may help to tailor the precipitation of fluoride and/or phosphate phases and, thus, ion concentrations in solution^13^, and (2) the phosphate/silicate ratio is strongly related with phase separation phenomena, not only in bioactive glasses but in glasses in general^14,15^. Phase separation may also determine the precipitation of crystalline phases upon immersion in biological solutions^16–18^. We aim not only to systematically investigate the effect of phosphate/silicate ratio in Na_2_O-free bioactive glasses on in vitro ion release and apatite precipitation upon immersion in Tris buffer, but to further visualise apatite mineralisation using transmission electron microscopy (TEM) with energy-dispersive X-ray spectroscopy (EDXS) and X-ray nano-computed tomography (nano-CT).

## Results and discussion

### Glasses before immersion in Tris buffer solution

Analysed fluoride contents have already been reported in our previous publication^19^, and phosphate/silicate ratio strongly influenced fluorine retention in the glass melts. The P_2_O_5_-free glass showed the lowest fluoride loss (13 ± 3%), which was associated with HF volatilization. Glasses P2, P3 and P5 lost 42 ± 19, 26 ± 7 and 55 ± 24% of fluoride, respectively**.** This difference we associated with an additional mechanism of fluoride loss in phosphate-containing compositions, involving the volatilization of phosphorus-containing fluoride compounds, e.g. POF_3_^19^.

**Figure 1:**
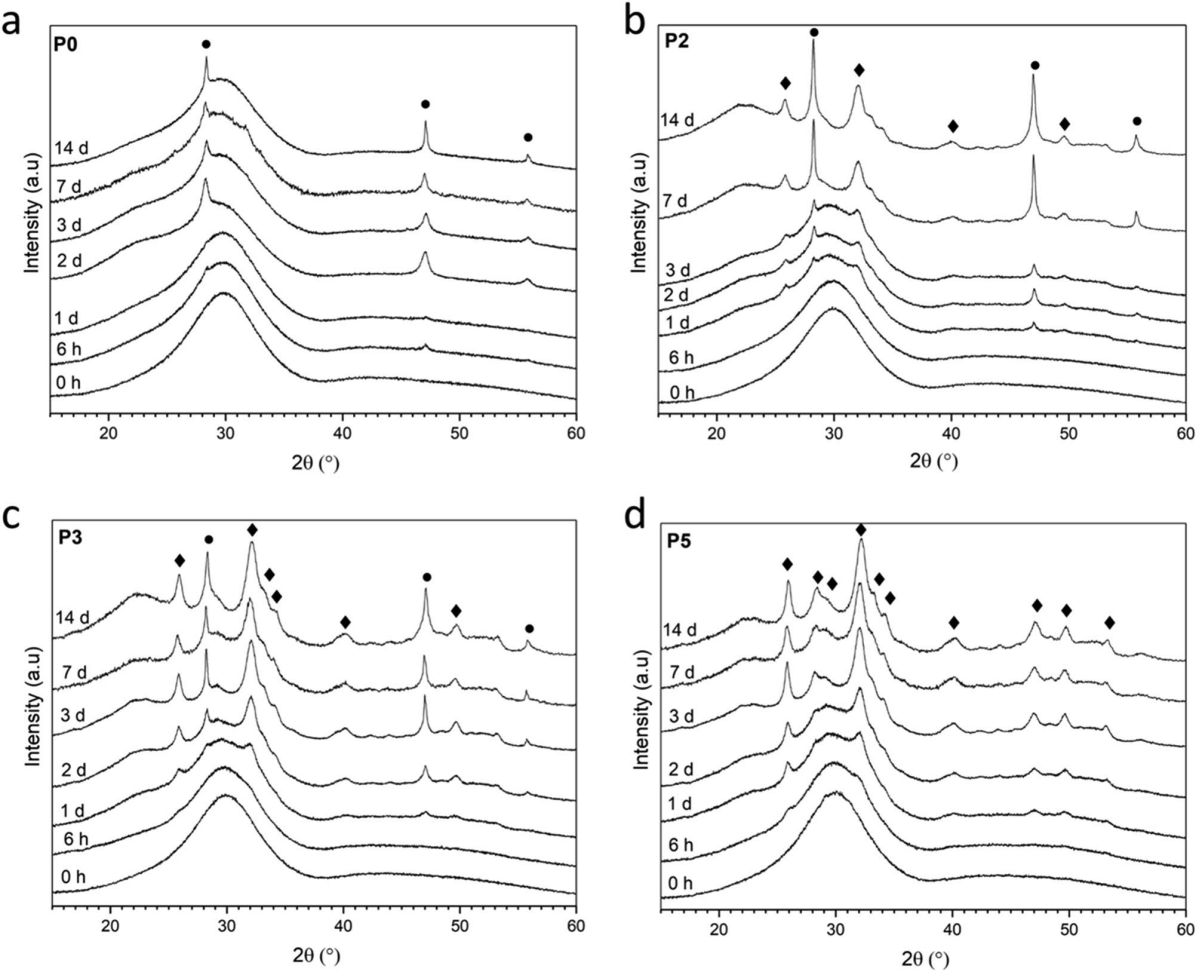
XRD patterns of glasses at various time points of immersion in Tris buffer solution. Circles correspond to fluorite, CaF_2_, and diamond shapes to fluorapatite, Ca_5_(PO_4_)_3_F.

**Figure 2:**
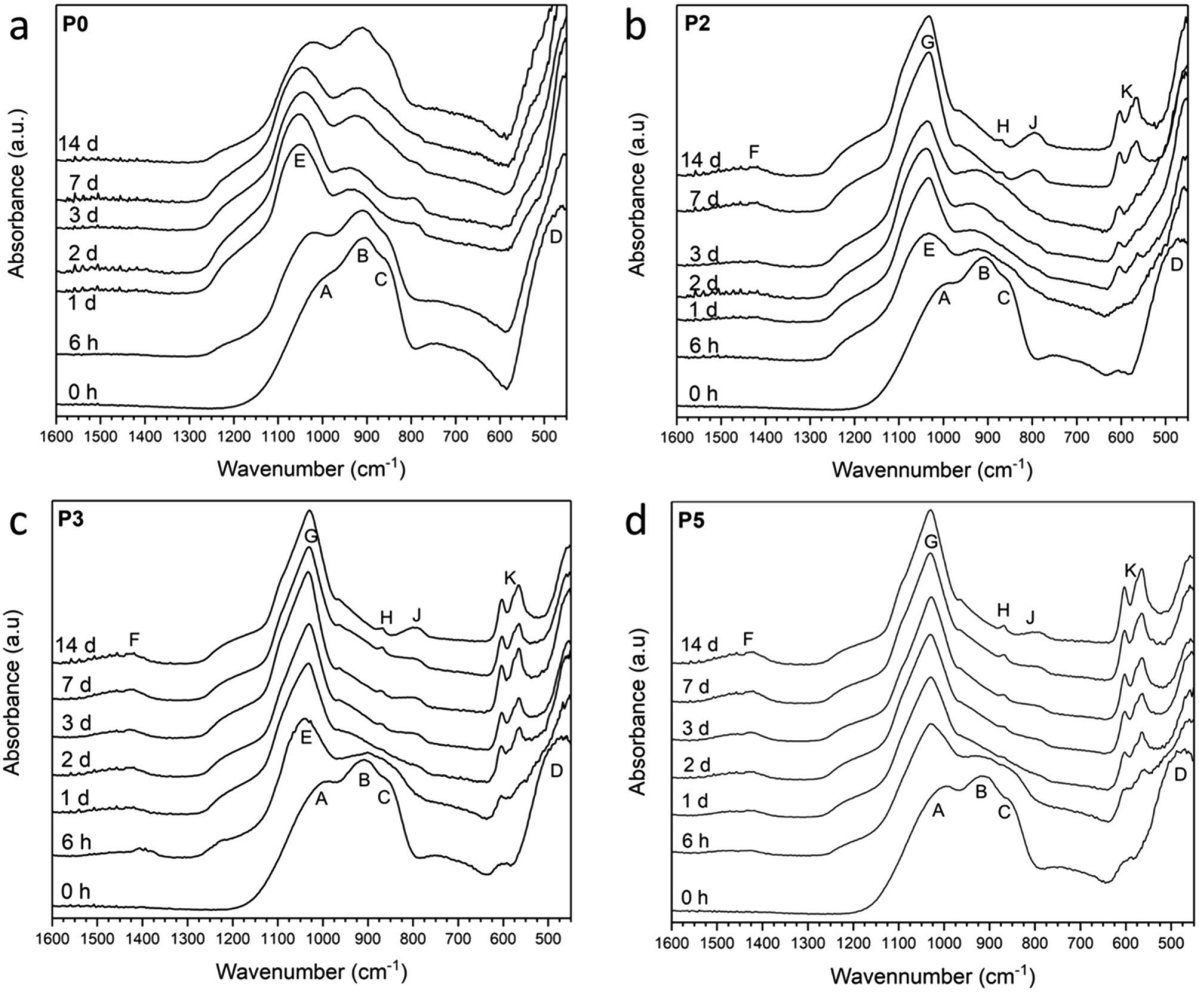
FTIR spectra of glasses at various time points of immersion in Tris buffer solution. Main bands are denoted from A to K (see text).

All glasses were amorphous according to X-ray diffraction (XRD) patterns (Fig. 1) showing the characteristic amorphous halo centred at approximately 30°2θ. Fourier-transformed infrared spectroscopy (FTIR) spectra (Fig. 2) show a broad band visible between 600 and 450 cm^−1^, which has been assigned to P-O and Si–O–Si bending vibrations^20^. The bands between 850 and 940 cm^−1^ (B,C in Fig. 2) correspond to *Q*^2^ groups with two non-bridging-oxygen atoms (NBO; ≡Si–O^−^), while the one positioned at about 1000 cm^−1^ (A) corresponds to *Q*^3^ (one NBO)^20^. No marked differences were observed with composition. The phosphate content in the present glass system was increased while adding stoichiometric amounts of calcium oxide, which maintains the number of NBO attached to phosphorus^21^. Therefore, the number of NBO attached to silicon and the silicon network connectivity remained constant throughout the series.

**Figure 3:**
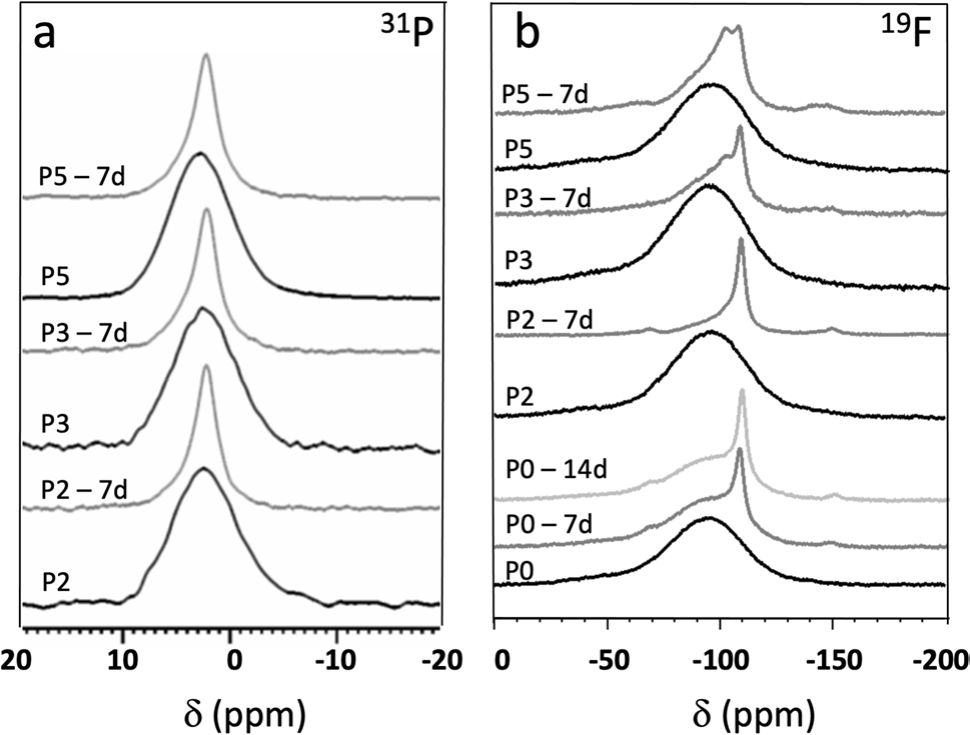
^31^P (**a**) and ^19^F (**b**) MAS NMR spectra of P2, P3 and P5 glasses before (black) and at 7 days immersion in Tris buffer solution (grey). ^19^F MAS NMR spectra of P0 glass before (black) and at 7 (grey) and 14 days of immersion (light grey) are also shown in (b).

Magic angle spinning nuclear magnetic resonance (MAS NMR) peaks observed for the untreated glasses (Fig. 3) are broad, in agreement with the low level of structural order typical of glasses. ^31^P MAS NMR spectra of untreated samples P2, P3 and P5 show a single peak at 2.5 to 2.8 ppm (Fig. 3a, Table 1), corresponding to orthophosphate (*Q*_*P*_^0^) groups charge-balanced by calcium ions, in agreement with other Na-free glasses reported in the literature^10^. The presence of a symmetric orthophosphate signal suggests absence or very low concentrations of Si–O–P bonds. Such direct chemical bonds between the silicate and the phosphate part of the glass structure are typically detected in molecular dynamics simulations^16^; however, the experimental side is less clear. While it might be possible that the concentration of Si–O-P bonds is too low to be detected by conventional 1D MAS NMR spectra, very detailed solid-state NMR studies on a ^29^Si and ^17^O-enriched sample of Bioglass 45S5 did not detect any Si–O–P bonds either^22^. By contrast, solid-state NMR studies on a calcium-free version of Bioglass 45S5 not only detected phosphate clustering but also found indications for the presence of Si–O–P bonds^23^. The occurrence of phase separation or clustering, such as presence of phosphate-enriched domains, in bioactive glasses has also been a matter of debate. Molecular dynamics simulations^16^ only found pronounced phosphate clustering for compositions much higher in phosphate content than 45S5, while a more recent study using dielectric measurements found two different sodium-hopping domains and interpreted this as caused by phase separation, most likely into a phosphate-rich and a silicate-rich phase^24^. TEM results can give insight into possible phase separation here, discussed further below.

**Table 1:** ^31^P and ^19^F chemical shift (ppm) before and at 7 days of immersion in Tris buffer solution (see MAS NMR spectra in Fig. 3).

Glass	δ(^31^P) before immersion	δ(^31^P) at 7 days of immersion	δ(^19^F) before immersion	δ(^19^F) at 7 (14) days of immersion	
P0	n/a	n/a	− 93.8	− 94.8 (− 94.8)	− 108.4 (− 108.8)
P2	2.50	2.24	− 96.5	− 101.0	− 108.6
P3	2.63	2.23	− 93.2	− 102.0	− 108.6
P5	2.80	2.21	− 96.4	− 102.0	− 108.1

Values at 14 days of immersion are also shown for P0 glass. For phosphate containing glasses, no analysis was carried out at 14 days as fluorapatite was already detected at 7 days of immersion.

^19^F MAS NMR spectra (Fig. 3b) also show single peaks only for untreated glasses, with chemical shifts varying between −93.8 (P0) and −96.4 ppm (P5), corresponding to an F-Ca(n) calcium fluoride-like environment. A single peak at −89 ppm was reported in a Na-free glasses of a similar composition^5,10^. It would be unlikely to have Si–F bonds, e.g., FCa_2_Si units, present, since their signals were reported to be positioned at −113.1 ppm^25^. It has been widely described that in bioactive glasses fluoride complexes modifier cations such as Ca^2+^ by ionic interaction and does not form Si–F bonds, as confirmed by ^19^F and ^29^Si MAS NMR spectroscopy^10,26,27^, X-ray photoelectron spectroscopy^26,28^, molecular dynamics simulation^25,29^ and other methods^26,30^. We thus expect fluoride here to be coordinated by calcium ions. Molecular dynamics simulations further suggested the formation or Ca-F rich clusters^26^, where calcium and fluoride species interact ionically, resulting in areas of low viscosity, prone to crystallisation.

**Figure 4:**
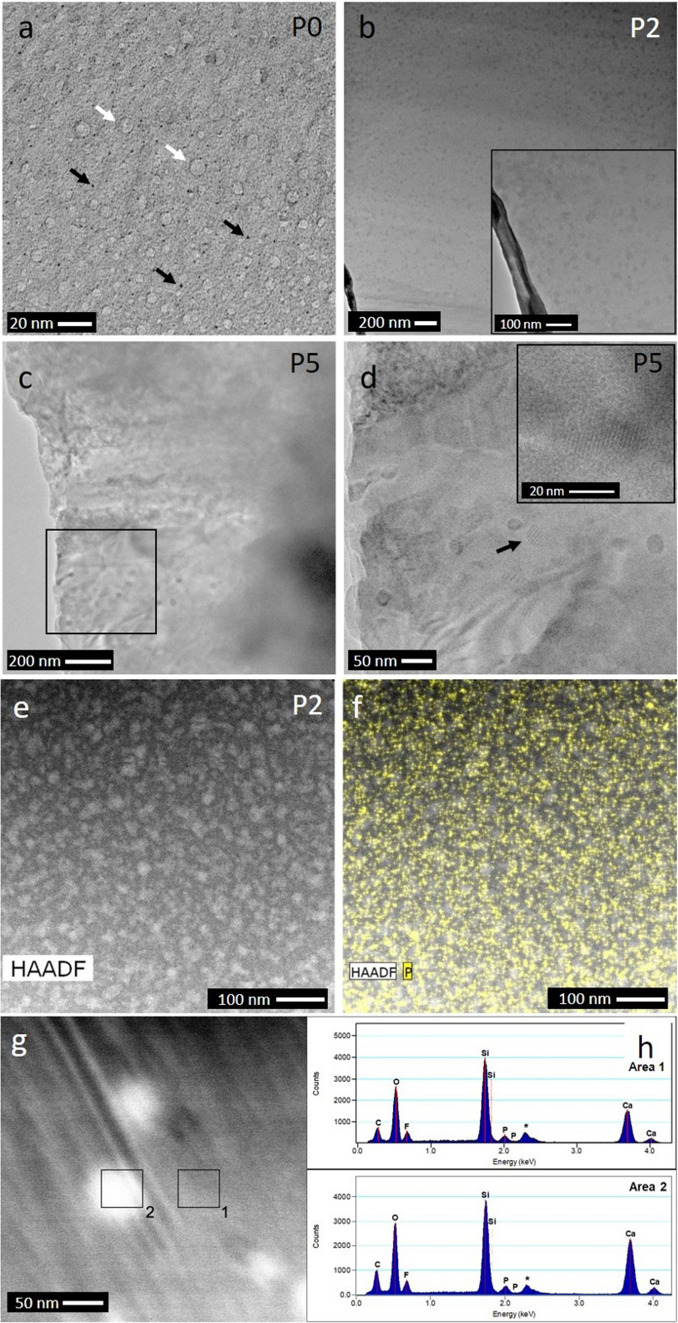
TEM bright field micrographs of glasses (**a**) P0 (bulk) (white arrows indicate electron radiation damage, and black arrows indicate PSD), (**b**) P2 (bulk) and (**c**,**d**) P5 (powder) (Figure d represents the area highlighted in a black rectangle in (**c**); black arrow indicates crystalline planes, also shown in the inset). (**e**) HAADF micrograph of P2 bulk and (**f**) same area with P distribution mapping superimposed. (**g**) STEM micrograph of the same P2 bulk glass sample as in (**e**,**f**) and (h) EDX spectra of areas denoted as 1 (matrix, top) and 2 (PSD, bottom) in (**g**). (*The EDXS peak at 2.3 keV corresponds to molybdenum from the TEM sample holder).

Glasses P0, P2 and P5 were further investigated using TEM-based techniques. Glasses P0 and P2 were imaged as bulk samples, while glass P5 was characterized in its powder form. In general, powder particles present an inhomogeneous thickness, and they are usually not electron transparent, which makes TEM interpretation difficult. This issue is overcome by bulk sample preparation for TEM analyses. Figure 4a shows the P0 microstructure before immersion. The larger roundish regions (white arrows in Fig. 4a) could correspond to electron radiation damage from the TEM. The dark areas of a few nanometres in diameter (black arrows in Fig. 4a) could be attributed to immiscibility phases or phase separation droplets (PSD). Since calcium is the highest atomic number element in these glasses, the dark colour in bright field images suggests that those PSD are enriched in Ca^2+^. This agrees with studies showing that fluorides can induce phase separation, in which the nucleation and crystallization takes place in the volume of the PSD^14,31–35^. Larger PSD were also observed in the phosphate-containing glasses (Fig. 4b–d), with those in glass P2 having irregular morphology. In glass P5, round features were observed at the thinnest part of the powder particles (Fig. 4c,d). In some areas, crystalline planes were discerned (black arrow and inset in Fig. 4d). However, these crystalline areas did not seem sufficient to be detected by XRD (Fig. 1, 2). Besides CaF_2_, it is known that P_2_O_5_ may induce phase separation^15,17^, and nanometre-sized Ca-P-rich clusters have been observed by TEM^18^. Based on this, the droplet phase in Fig. 4 is likely to be enriched in P, Ca, and F, while the matrix phase is rich in silicon.

EDXS analysis was performed on glass P2 (2.4 mol% P_2_O_5_). In the HAADF image (Fig. 4e) irregular areas are observed as bright contrast, which indicates an enrichment in the element of highest atomic number (Z-contrast), which is Ca. Figure 4h displays EDXS results of one of those bright areas and of the matrix. Both show very similar elemental concentrations, with only slightly higher calcium concentrations in the bright droplet. This similarity in chemical composition suggests that the bright contrast in the micrograph (Fig. 4g) may have a second contribution from diffraction contrast in addition to the Z-contrast. Diffraction contrast originates from differences in the orientation of the atomic layers rather than density or chemical composition (similar to what we observed previously for Bioglass 45S5^36^). Both contrast contributions can be explained as very early stages of crystallisation, i.e. precursors of crystalline phases with an atomic medium-range order, possibly involving calcium fluoride-like areas^10,37^, owing to the slightly higher concentration in calcium. Pre-ordered fluoride bonds have been determined by ^19^F MAS NMR in glasses containing BaF_2_ (Ba-F bonds^38^) and LaF_3_ (La–F bonds^39^). The phosphorus distribution mapping is superimposed in yellow (Fig. 4f). P is heterogeneously distributed, but clear enrichment areas are not observed for this glass. This suggests clustering rather than phase separation, despite molecular dynamics simulations predicting phosphate clustering for higher concentrations of phosphate only^16^.

### Changes during immersion in Tris buffer solution

**Figure 5:**
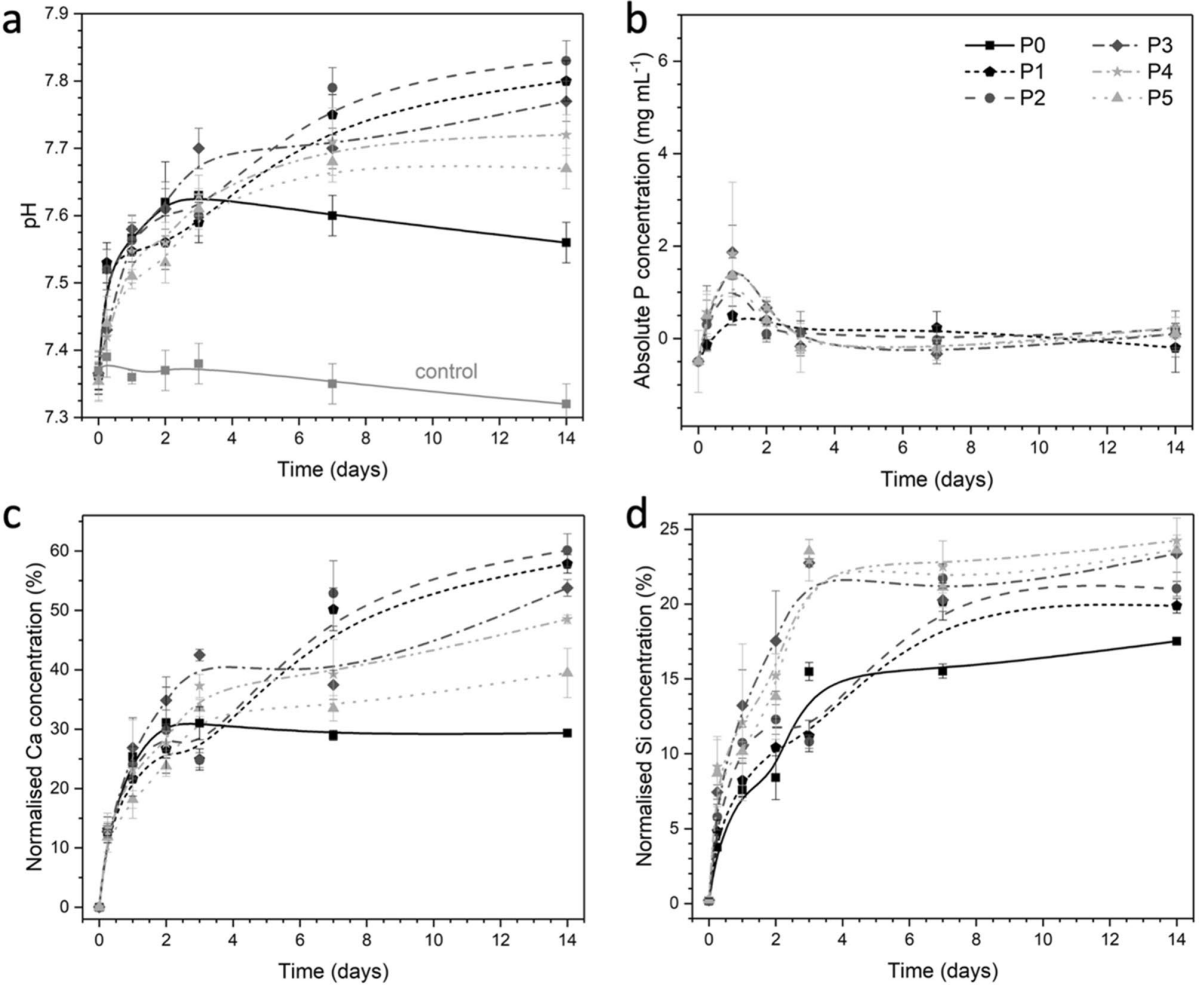
Change of (**a**) pH, (**b**) phosphorus, (**c**) calcium and (**d**) silicon concentration in Tris buffer over time. Calcium and silicon concentrations are presented as normalised to the respective nominal ion content in the glass.

All glasses caused an increase in the pH of the Tris buffer solution (Fig. 5a), which is characteristic for bioactive phospho-silicate glasses and is caused by a rapid ion exchange between modifier cations (here, Ca^2+^ only) attached to NBO and protons from the solution^17^. This pH increase is very fast at early time points and slows down with time, particularly for glass P0, which seems to give a constant pH from day 3 (the slight pH decrease observed mirrors that of the glass-free control). At 14 days, pH values in solution seem to decrease with phosphate/silicate ratio in the glass (Fig. 5a,c), an effect which has been reported earlier^9^. This relationship between pH rise in solution and phosphate content in the glass can be explained with the pH rise originating from the ion exchange between the silicate part of the glass, as modifier cations (ionically connected to NBO) are released in exchange for protons. By contrast, orthophosphate groups and calcium ions charge balancing them are released together^7^. Phosphate-free composition P0 is showing the lowest pH rise and the lowest relative calcium concentration in solution. A possible explanation may be less water intrusion in this composition, owing to the absence of phosphate groups and, as a result, a more densely packed glass network. The release of calcium and fluoride ions from the Ca-F environment in the glass does not affect the pH either^37,40^. The less prominent increase in pH from day 7 could be associated with the decrease in ion release, which would be obtained by depletion of the ions and the formation of a passivating apatite layer as discussed below.

A common feature in all glasses here is that ion release is fastest within the first 3 days. Such rapid release is typical for bioactive glasses, has been observed in dynamic ion release studies at very early time points^41^, and it is explained by a highly disrupted silicate network allowing for fast water intrusion and ion exchange to happen^42,43^. To facilitate comparison between glass compositions, calcium and silicon ion concentrations in solution were normalised to the content of the respective ion in the glass, i.e., they are shown as relative concentrations in percentages. Curves of calcium concentrations in Tris buffer over time mirror the pH curves. pH changes during immersion of bioactive glasses can be described as a summation of all ion exchange occurring between modifiers attached to NBO and the solution^44^, and calcium ions are the only modifier ions in the present glass system. However, ion concentrations detected in solution depend on both ion release from the glass and precipitation of solid phases from the solution and, as discussed below, precipitation of two calcium-containing phases occurs in the present system. A likely explanation for the matching trends in pH and calcium concentrations is that precipitation consumes stoichiometric amounts of calcium associated with phosphate and fluoride in the glass, while calcium ions remaining in solution correspond roughly to the stoichiometric amounts of modifiers attached to NBO. Silica-species have a low solubility in aqueous solutions of the physiological pH conditions studied here, with the actual solubility showing pH dependence^45^. Except for composition P0, concentration of silica species (Fig. 5d) shows an inverse relationship with pH, which may simply reflect this relationship of silica solubility with pH in this range.

Besides pH trends and ion concentrations, ion exchange is also reflected in FTIR spectra and XRD patterns^46^. The intensity of bands B and C corresponding to NBO (ca. 850 to 940 cm^−1^) decreases drastically at early time points (Fig. 2). For P0, this effect is apparent from 1 day of immersion, while for the other glasses the bands have virtually disappeared at 6 h. The corresponding appearance of an additional amorphous halo in XRD patterns (Fig. 1) at lower 2θ values illustrates the formation of an ion-depleted layer, typically referred to as silica gel layer^47,48^. FTIR band J appearing at 790 cm^−1^ also reflects silica gel formation, corresponding between vibrations between SiO_4_ tetrahedra^20^. Besides the formation of an ion-depleted glass layer, precipitation of crystalline phases has been observed with apatite being of particular interest. Phosphate is typically the limiting factor for apatite formation, which the present results confirm (Fig. 5b). As immersion experiments here were performed in a medium free of phosphate (as well as calcium and fluoride) ions, phosphate content in and release from the glasses directly influences precipitation. FTIR spectra of P0 (Fig. 2a) show no indication of precipitation apart from silica gel formation; XRD patterns (Fig. 1a), by contrast, show precipitation of calcium fluoride (CaF_2_, ID 00-035-0816) from 2 days of immersion, which is also reflected in ^19^F MAS NMR spectra (7 and 14 days, Fig. 3b) showing narrow peaks positioned at about −108 ppm (Table 1)^49,50^. The broad feature still present at about −94 ppm indicates that there is still a contribution from amorphous F-Ca(n) species in the glass^5,10^.

It is interesting to note that the split FTIR band between 560, 580 and 600 cm^−1^ (K) appears at early time points, with spectra showing no indication of amorphous CaP being present (except maybe for P2 at 6 h, Fig. 2b). A possible explanation is the relatively simple glass composition, containing only calcium ions as modifiers, and the effect of fluoride ions accelerating apatite precipitation^51^ by preventing the formation of octacalcium phosphate as an intermediate step^52^. This early apatite formation is also reflected in results on ion concentrations in solution. Phosphate concentrations from 6 h in solution were very low in agreement with previous studies^6,53^, and this result has been associated with a rapid phosphorus release before the first time point of analysis causing early apatite precipitation which subsequently resulted in the solution being depleted in phosphate species^53^. In the present study, phosphorus concentrations were around the detection limit except for glasses P3 to P5 at 6 h and 1 day (Fig. 5b). These slightly higher concentrations for P3 to P5 suggest that phosphate availability was not yet the limiting factor for apatite formation here, and that depletion occurred later.

The apatite formed on bioactive glasses is often described as biomimetic owing to its nano crystallinity^54^ and its carbonate substitution. Presence of carbonate substitution is indicated by the band at about 870 cm^−1^ (band H in Fig. 2b–d; C–O stretch) as well as broad bands between 1400 and 1600 cm^−1^ (F), with the position of the latter indicating B-type substitution, i.e. carbonate replacing a phosphate band^55^.

### Micro- and nano-structural changes during immersion in Tris buffer solution

**Figure 6:**
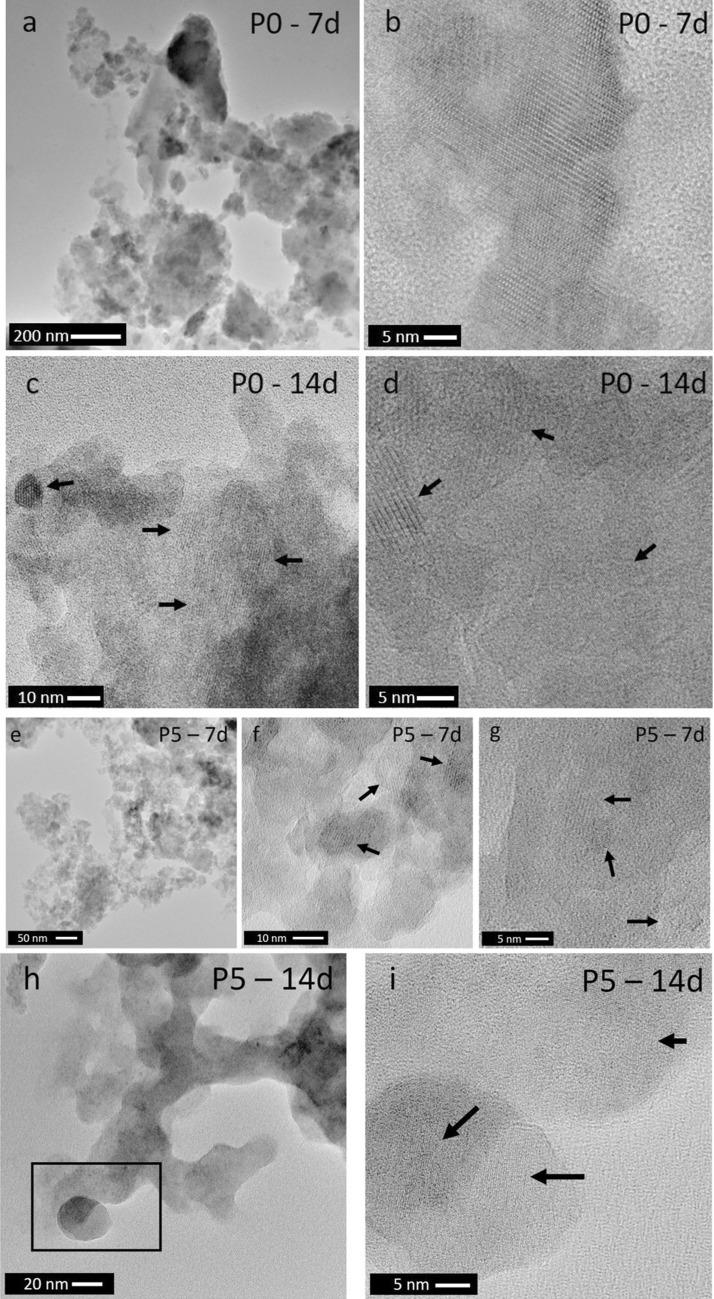
TEM micrographs of P0 powder at (**a**,**b**) 7 and (**c**,**d**) 14 days of immersion in Tris buffer solution; (b, c, d) display details of crystalline planes. TEM micrographs of P5 glass powder at (**e**–**g**) 7 and (**h**,**i**) 14 days of immersion in Tris buffer solution. (Figure (**i**) represents the area highlighted in a black rectangle in Figure h; black arrows indicate crystalline planes).

Figure 6a,b shows sub-micron scale TEM micrographs of the P0 powder microstructure at 7 days of immersion in Tris buffer solution. In addition to homogeneous areas, agglomerates of smaller particles were also observed. Crystalline planes were clearly visible at higher magnification, which according to the XRD results (Fig. 1) can be attributed to CaF_2_. At 14 days of immersion, the coarse morphology of the powder particles does not change significantly. However, the crystalline area covers a more extensive area (black arrows in Fig. 6c,d). Owing to the absence of phosphate ions in glass and immersion medium, no precipitation of calcium phosphate (CaP) species can possibly occur for composition P0.

With increasing phosphate content in the glass, the situation changes. From 1 day of immersion, FTIR spectra of immersed P2 (Fig. 2b) show a split band (K) at 560, 580 and 600 cm^−1^, which is characteristic for apatitic orthophosphate groups^55^. Concomitant appearance of the narrow P—O stretch band positioned at 1030 cm^−1^ (G) further confirms the beginning of apatite crystallisation. For glasses P3 and P5 (Fig. 2c,d) these bands are visible from 6 h of immersion already, showing that the higher phosphate content in the glass caused larger or faster phosphate release (further discussed below) and, subsequently, faster apatite precipitation. For glasses P2 to P5, XRD patterns (Fig. 1b–d) show appearance of apatite formation later than FTIR spectra (which is not surprising as ATR-FTIR is more surface sensitive than XRD), but they confirm that, indeed, apatite forms (e.g. fluorapatite, Ca_3_(PO_4_)F, ID 01-071-0880, or a partially fluoride-substituted apatite). In addition, they show reflections corresponding to calcium fluoride for P2 and P3. ^31^P MAS NMR spectra (Fig. 3a) further confirm apatite formation: while the peak positions before and after immersion (Table 1) have changed slightly only, the linewidth has narrowed drastically for spectra after immersion, indicating a more ordered structure around phosphorus atoms, i.e. a crystalline calcium orthophosphate environment. ^19^F MAS NMR spectra of glasses P2 to P5 at 7 days' immersion (Fig. 3b) show a signal at −101 to −102 ppm, associated with fluorapatite^56^, which was weak only for P2 but increased in intensity with phosphate content in the glass. This signal appeared as a dominant feature for P5, confirming that the apatite formed is fluorapatite.

**Figure 7:**
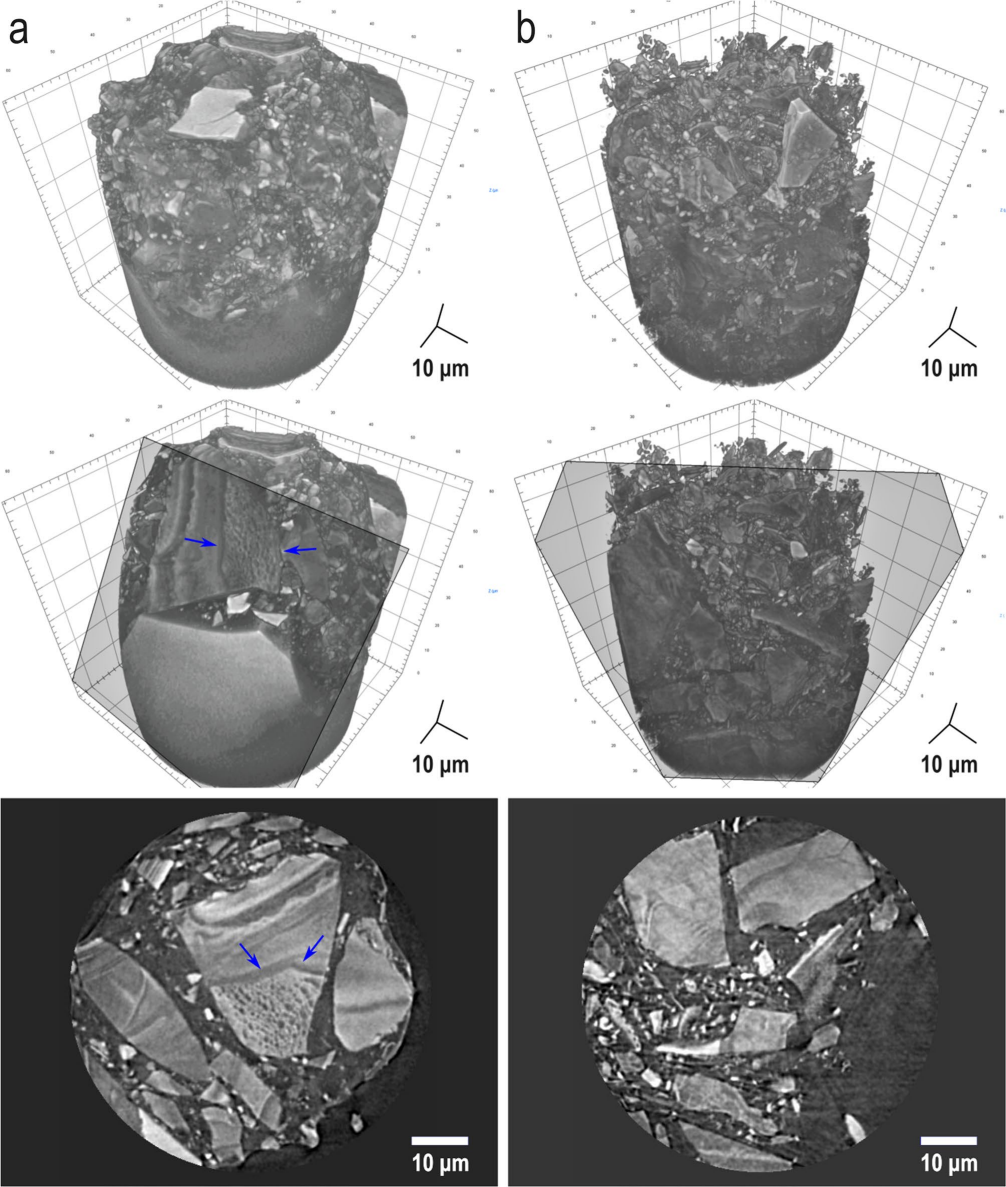
Nano-CT images of samples (**a**) P0, (**b**) P2 at 14 days of immersion in Tris buffer. Volumetric reconstructions of the samples (top) with a virtual cut (centre) and a tomogram (bottom).

Using nano-CT, we investigated whether changes in glass morphology had occurred during immersion in Tris buffer. The three-dimensional nano-CT images of P0 and P2 powder samples at 14 days of immersion in Tris buffer (Fig. 7) show the morphology of the samples and the distribution in space of various particles with irregular shapes and different sizes, ranging from some tens of micrometres to hundreds of nanometres. The larger and rather edgy particles also show different surface textures, appearing either smooth or rough due to the presence of pores of some hundreds of nanometres (Fig. 7a, blue arrows). Although we imaged the samples by Zernike phase-contrast, the grey level distribution of the images also carries some absorption-contrast information, and the pixel grey level and the electronic density of the chemical elements in the sample are related. Therefore, the domains below 5 µm in size, which are smaller and more rounded and show a different grey level than the matrix (which is mainly composed of silicon) could be related to the presence of calcium. Indeed, the crystallisation of CaF_2_ in P0 (Fig. 7a, bottom) and CaF_2_ and fluorapatite in P2 (Fig. 7b, bottom) correlate well with the presence of these brighter particles in nano-CT images and also support the results obtained by EDX.

**Figure 8:**
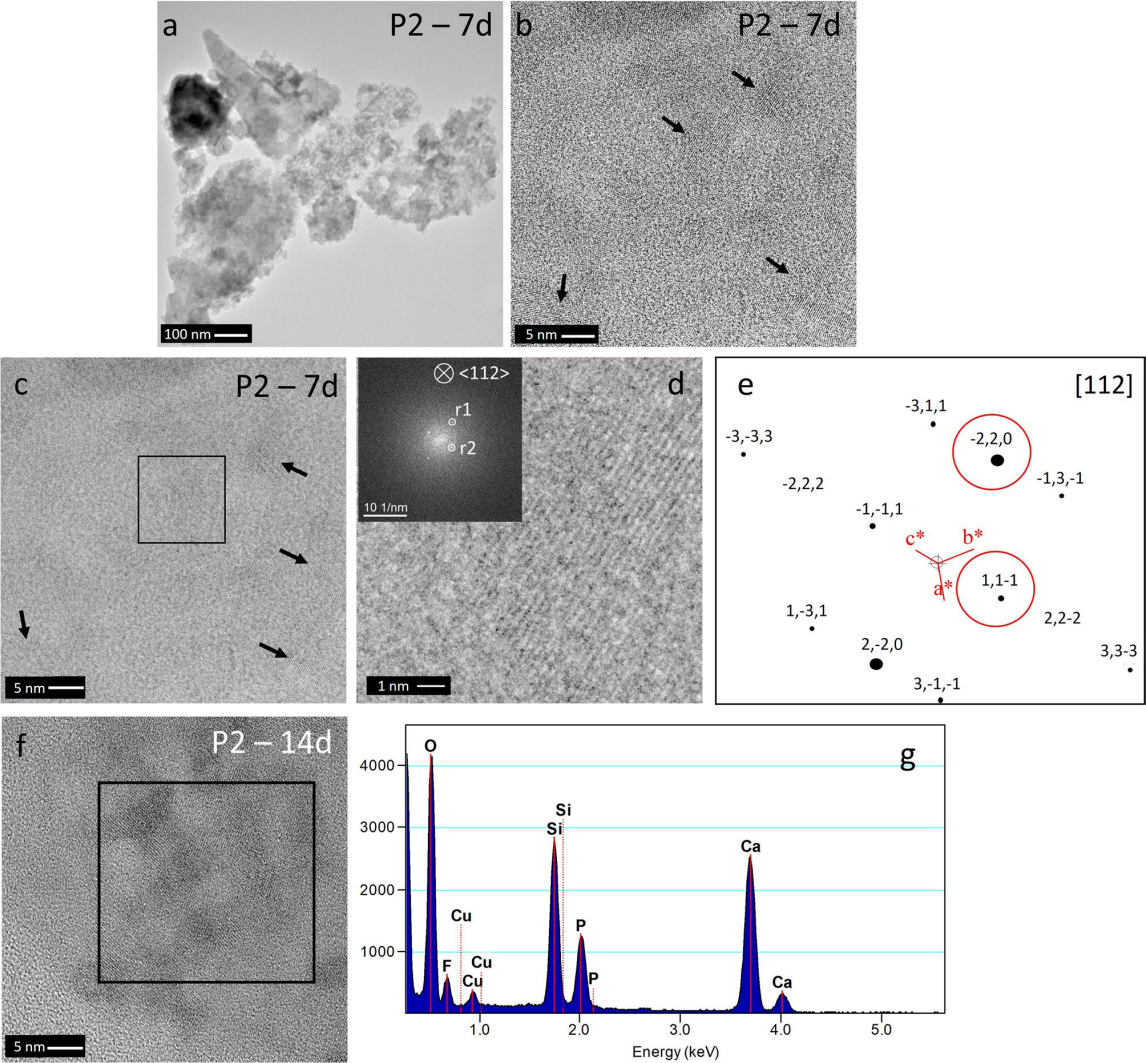
(**a**,**b**) TEM micrographs of P2 powder at 7 days in Tris buffer (black arrows indicate crystalline planes), (**c**) HRTEM micrograph with marked crystalline area for FFT analysis, (**d**) region of interest (ROI) with corresponding power spectrum and indexed spots for d-spacing calculation, (**e**) calculated EDP from XRD data of CaF_2_ (ICSD 28730) with [112] viewing direction and matching reference spots highlighted, (**f**) TEM micrograph of the P2 glass powder at 14 days in Tris buffer and (**g**) EDXS spectrum of the crystalline area marked in a black box.

Figure 8 shows the micro- and nanostructure of P2 powder at 7 and 14 days of immersion, showing similarities to those seen for P0 (Fig. 6a,b). At higher magnifications, small crystalline areas are densely distributed in the matrix (Fig. 8b). Moreover, from the HRTEM image (Fig. 8c) a power spectrum analysis and fast Fourier transform (FFT) of the crystalline area (Fig. 8d,e) show distinctive spots r1 and r2 which can be correlated with the r(−220) and r(11–1) positions of the calculated electron diffraction pattern based on CaF_2_ (ICSD 28,730), confirming crystallization of CaF_2_ in that particular area of sample P2 at 7 days. EDXS analysis performed at 14 days of immersion (Fig. 8f,g) shows the following composition (in at%): 37.4 Si, 38.0 Ca, 8.6 F and 16.0 P, which matches that of fluorapatite (with ratio Ca:F being nearly 5:1 and Ca:P nearly 5:3). Figure 6e-i shows P5 powder microstructure at 7 and 14 days of immersion. Crystalline planes are observed at 7 days (Fig. 6f,g), and crystalline areas are located at the edges of the powder particles (Fig. 6e), which corresponds to the crystalline phases detected by XRD analysis.

Taken together, TEM highlights the presence of phosphate clustering and nearly crystalline calcium fluoride environments in SiO_2_-P_2_O_5_-CaO-CaF_2_ glasses, which are likely to affect not only ion release and precipitation but possibly also crystallisation at elevated temperatures. By using a combination of various analytical methods, we can illustrate how ion release is followed by surface precipitation, and how this process changes if the phosphate/silicate ratio in the glass increases. Nano-CT and TEM made it possible to visualise how these changes affect the bioactive glass morphology and microstructure at a sub-micron to nanometre-level after mineralisation.

## Methods

### Glass design and preparation

Glasses in the system SiO_2_-P_2_O_5_-CaO-CaF_2_ (Table 2) were designed by increasing the phosphate/silicate ratio while maintaining the silicate network polymerisation (network connectivity, NC = 2.11)^7,17^ comparable to that of Bioglass 45S5. Glasses were named based on their P_2_O_5_ content.

**Table 2:** Nominal glass compositions (mol%) and P_2_O_5_/SiO_2_ ratio.

Glass	SiO_2_	P_2_O_5_	CaO	CaF_2_	Ratio P_2_O_5_/SiO_2_
P0	46.8	0	44.2	9.0	0
P1	44.3	1.2	45.5	9.0	0.03
P2	41.9	2.4	46.7	9.0	0.06
P3	39.9	3.4	47.7	9.0	0.09
P4	38.0	4.3	48.7	9.0	0.11
P5	36.3	5.1	49.6	9.0	0.14

Glasses were melted in platinum–rhodium crucibles; 150 g batches contained mixtures of analytical grade SiO_2_ (99.0%, Carl Roth, Germany), Ca(H_2_PO_4_)_2_·2H_2_O (Budenheim KG, Germany), CaF_2_ (Chemiewerk Nünchritz, Germany), and CaCO_3_ (≥ 99.0% Merck, Germany). The raw materials were melted in an induction furnace (in-house built) at temperatures of up to 1550 °C. Glasses P0-P3 were cast into brass moulds. Glass transition temperatures (T_g_) were determined by differential scanning calorimetry (STA 449 F1 Jupiter, NETZSCH-Gerätebau, Germany) at 10 K/min heating rate. Based on the T_g_ values around 653 °C the glasses were annealed at 670 °C (± 5 K) for 2 h. Compositions P4 and P5 had a high tendency to devitrify and were therefore quenched only without subsequent annealing. The glasses were crushed using a metallic mortar, sieved (125 to 250 µm) and stored in a desiccator. Glasses P0, P2 and P5 were chosen for imaging characterisation. Bulk pieces of P0 and P2 were prepared by drilling rods (5 mm in diameter) from the annealed glass blocks and subsequently cutting into 5 mm thick pieces with a low-speed diamond saw (Struers Minitom, Denmark) and low viscosity oil. Glass P5 was only characterized as powder since it was not possible to obtain bulk pieces owing to its inherent devitrification tendency. Fluoride quantification was carried out by Laser-induced breakdown spectroscopy as detailed previously^19^.

### Immersion experiments

For in vitro dissolution tests, 75 mg of glass powder (125 to 250 µm particle size range) was immersed in 50 mL of 0.062 mol L^−1^ Tris buffer solution (pH 7.4) following a previously described protocol^46^. Samples were placed in an orbital shaker at 37 °C and 100 rpm for 6, 24, 48, 72 h, 7 or 14 days. Experiments were performed in triplicates. At each time point, the pH was measured using a pH electrode and meter (S40 SevenMulti™, Mettler Toledo) and samples were filtered; leachates were acidified using 1 M HNO_3_ solution and analysed by inductively-coupled plasma optical-emission spectroscopy (ICP-OES; 5110, Agilent Technologies, Germany). Glass powders were analysed as described in the following.

### Powder characterisation

Samples before and after immersion in Tris buffer solution were characterised by X-ray diffraction (Rigaku Miniflex 300, Cu K_α_, 15 kV and 40 mA). The powders were milled finely for analysis, and patterns were recorded with a scan range between 10 and 60°2θ and a 0.02° step size. Phase identification was carried out using PDXL software together with the ICDD PDF-2 database.

Attenuated total reflectance Fourier transformed infrared spectroscopy (ATR-FTIR; Cary 630, Agilent Technologies, Germany) in absorbance mode was used to further examine the powders. For analysis, powders were ground with an agate pestle and mortar, and spectra were collected between 4000 and 400 cm^−1^ with a resolution of 4 cm^−1^ and 16 scans per measurement. An initial background scan was carried out before each set of samples.

Magic angle spinning nuclear magnetic resonance spectroscopy was used to study the phosphorus (^31^P) and fluorine (^19^F) chemical environment in the glasses before and after immersion. These analyses were performed on a Bruker Avance 400 Ultrashield spectrometer (Bruker BioSpin) operating at a 14.1 T magnetic field, and data processing was carried out using TopSpin. Glass powders were packed in a 2.5 mm rotor and rotated at 12 kHz and 22 kHz for ^31^P and ^19^F, respectively, with 32 scans per analysis. ^19^F chemical shift was referenced using the −120 ppm peak of 1 M NaF aqueous solution as a secondary reference against CFCl_3_; ^31^P chemical shift was referenced to 85% H_3_PO_4_ solution (0 ppm).

### Imaging techniques

Transmission electron microscopy was employed to characterize samples P0, P2 and P5 before and after immersion in Tris buffer solution. Bulk sample preparation (P0 and P2) was carried out by wedge-polishing (Allied High Tech MultiPrep, USA), followed by double-sided ion-beam milling using low-energy (2.5 keV) argon ions under a small angle of incidence (6°) until grinding and polishing artefacts were removed (precision ion-polishing system, PIPS + II, Gatan, Inc.). Electron-transparent areas of the sample were selectively coated with carbon using a special coating mask^57^ prior to TEM investigation to reduce charging effects resulting from interaction with the electron beam. TEM powder samples (P5 before immersion, and P0, P2 and P5 glasses after immersion) were suspended in ethanol. A droplet of the dispersion was dried on a carbon-coated copper grid and mounted in the TEM sample holder. High resolution (HR) bright field images and scanning transmission electron microscopy (STEM) images were recorded in a TEM microscope (FEI Tecnai G2 F20, Thermo Fisher Scientific, Netherlands) operating at 200 kV and camera length 200 mm.

Energy dispersive X-ray spectroscopy (EDXS) area analyses of Si (K-line), P (K-line), Ca (K-line) and F (K-line) were recorded in the same microscope (percentage of oxygen was calculated as difference to 100%). The bulk TEM sample of glass P2 before immersion was additionally investigated in a more advanced TEM microscope (FEI Titan 80–300, Thermo Fisher Scientific, Netherlands) with spherical aberration corrector and high-brightness gun at 80 kV acceleration voltage and 70 mm camera length. High-angle annular dark field (HAADF) images and EDXS elemental distribution mappings were obtained to gain information about elemental distribution. The EDXS detector (Super X, Thermo Fisher Scientific, Netherlands) consists of four silicon drift detectors, offering a maximum collection angle of 0.8 sr. The analytical accuracy of the EDXS measurement was assessed to be around 2%.

In the powder sample of sample P2 at 7 days of immersion in Tris buffer solution, fast Fourier transformation (FFT) of HRTEM images (also called a power spectrum) was obtained (Digital Micrograph software, Gatan). Measured d-spacings calculated from the power spectra were compared with reference d-spacings of specific candidates (Ca,F) preselected by XRD evaluation. Structural data, i.e. atomic positions of the references, were taken from the Inorganic Crystal Structure Database (ICSD, Fachinformationszentrum Karlsruhe, Germany, and the National Institute of Standards and Technology, USA). The structures were then rebuilt using CaRIne Software (CaRIne Crystallography 3.1) to generate d-spacing lists and simulate the electron diffraction pattern (EDP).

Samples P0 and P2 were imaged using a Carl Zeiss Xradia 810 Ultra X-ray microscope equipped with a chromium source (5.4 keV). The powdered samples were imaged inside a polyimide tube (Goodfellow Cambridge Ltd., LS522958) glued onto the tip of a metallic pin that was inserted in the sample holder. The samples were scanned using Zernike phase contrast, a field-of-view of 64 µm^2^ with a detector binning of 2 (isotropic pixel size 128 nm^2^) and a total of 901 projection images, each one with an exposure time of 50 to 60 s. These were acquired by rotating the sample 180° and the drift was corrected using the adaptive motion compensation from the software. Image reconstruction for obtaining the X-ray nano-computed tomography (nano-CT) dataset was performed by a filtered back-projection algorithm using XMReconstructor software. The tomograms obtained were then exported as a stack of 16-bit TIFF images. Vision4D software (arivis AG) was used for the volumetric visualisation.
